# Prevalence of vascular complications and factors predictive of their development in young adults with type 1 diabetes: systematic literature review

**DOI:** 10.1186/1756-0500-7-593

**Published:** 2014-09-02

**Authors:** Steven James, Robyn Gallagher, Janet Dunbabin, Lin Perry

**Affiliations:** Huntsville District Memorial Hospital, Muskoka Algonquin Healthcare, 100 Frank Miller Drive, Huntsville, Ontario P1H 1H7 Canada; Charles Perkins Centre/Sydney Nursing School, University of Sydney, Sydney, New South Wales 2006 Australia; University of Newcastle, University Drive, Callaghan, New South Wales 2308 Australia; Faculty of Health, University of Technology, Sydney, 15 Broadway, Ultimo, New South Wales 2007 Australia

**Keywords:** Quantitative epidemiological systematic review, Vascular complications, Prevalence, Predictors, Retinopathy, Nephropathy, Hypertension, Young adults, Type 1 diabetes

## Abstract

**Background:**

Vascular complications curtail life expectancy and quality of life in type 1 diabetes and development at younger ages is particularly detrimental. To date no review has summarised the prevalence or factors predicting their development in young adults.

**Methods:**

A quantitative epidemiological systematic review was conducted to identify the prevalence and predictive factors for development of retinopathy, nephropathy and hypertension in young adults (sample age mean [plus 1SD] 18–30 years) with type 1 diabetes, using processes adapted from established review methods set out by the Centre for Reviews and Dissemination.

MEDLINE (Ovid), Scopus (Elsevier), CINAHL, Science Direct (Elsevier), Google Scholar and Cochrane databases were searched to identify relevant articles published between 1993 and June 2014. From this eleven papers were retrieved, appraised and results summarised by three reviewers using established methods.

**Results:**

Some form of retinopathy occurred in up to almost half of participants; more severe forms affected up to one in ten. One in six was reported with microalbuminuria; one in 14 had macroalbuminuria. Hypertension occurred in almost one in two participants. Applying out-dated high thresholds this decreased to approximately one in ten participants. Glycaemic control was a consistent predictor of vascular disease in this age group.

**Conclusion:**

Prevalence rates of retinopathy, nephropathy and hypertension in young adults with type 1 diabetes emphasise the importance of regular complication screening for early detection and treatment. The predictive effect of glycaemic control reinforces its importance for prevention of vascular complications.

**Electronic supplementary material:**

The online version of this article (doi:10.1186/1756-0500-7-593) contains supplementary material, which is available to authorized users.

## Background

The increasing incidence of type 1 diabetes in many countries challenges health systems because the disease is presently incurable with no known method of prevention [1]. Around 490,100 children live with the disease worldwide, with incidence estimated to be increasing in children under 15 years by 2.8% per year [1, 2]. This trend is particularly worrying because type 1 diabetes increases mortality and morbidity population-wide, including in young adults [3–5]. People with type 1 diabetes diagnosed before the age of 30 years have been calculated to have a 4.7-fold excess mortality risk [6].

Vascular complications are often the cause of this early mortality. However, there is currently little information available about the prevalence or predictive factors for development of vascular complications of type 1 diabetes among young adults. Identification of disease complication prevalence and any predictive characteristics will establish a benchmark of these risk factors, and may assist healthcare professionals to target appropriate information and support with the aim of deferring or averting their onset.

The development of vascular disease in type 1 diabetes has been proposed as a consequence of disordered activity of lipid metabolism enzymes or transporters affecting endothelial function, inflammation, coagulation, platelet activation and fibrinolysis [7]. In combination with population-wide cardiovascular and athero-thrombosis risk factors, a state of persistent and progressive damage to the vascular wall (macro-angiopathy) is created [8]. Micro-angiopathic disease also occurs, with hyperglycaemia a leading pathogenic factor [9]. Vascular co-morbid diseases include retinopathy, which may cause reduced vision and blindness, and nephropathy, which may result in renal failure and require dialysis or kidney transplantation. This is in addition to hypertension, which is linked to peripheral, cardio- and cerebrovascular disease, the end points of which are limb amputations, cardiac failure, stroke and sudden death. As vascular complications curtail both life expectancy and quality of life [10], development at younger ages when people are typically establishing careers and families is particularly detrimental.

Young adults may be particularly vulnerable to complications because many have unique health needs relating to their psychological, physical and socio-cultural life stage issues; these commonly lie outside health services’ remit and place them at high risk for poor self-management. Diabetes services are predominantly structured into exclusive paediatric and adult services, with transition between these services not clearly the responsibility of either: an arrangement which serves young adults poorly. This transition stage was first formally identified as challenging by Blum et al. [11], however, decades later similar levels of difficulty with the transition process are still being reported [12]. Consequently, young adults with type 1 diabetes may not adhere to diabetes regimes, and may disengage from diabetes services after transition [13, 14]. Attrition from, or failure to engage with, diabetes services as an adult too-frequently results in reduced diabetes self-management and well-being, and inadequate screening for complications.

The potential benefits of models of transition to maintain engagement with adult diabetes services post-transfer from paediatric care have been flagged, and characteristics associated with reduced attrition and increased satisfaction and successful service redesign described [15–17]. However, the effect of transition service redesign has not been examined in terms of outcomes such as onset of vascular complications; neither has there been any attempt to summate or quantify the degree of complication-related morbidity and early mortality experienced by this young adult group. Lack of international benchmarks limits evaluation and deters appropriate prioritisation of service redesign to promote retention of young adults in contact with services, an essential element in achieving good glycaemic control to defer onset of complications [18–20].

The aim of this review was to identify the prevalence and factors predictive of development of vascular complications (retinopathy, nephropathy and hypertension) occurring in young adults with type 1 diabetes. For the purpose of this review, the term young adult refers to ages 18–30 years inclusive.

## Methods

A quantitative epidemiological systematic review was conducted using processes adapted from established review methods set out by the Centre for Reviews and Dissemination [21]. Standards derived from the Preferred Reporting Items for Systematic Reviews and Meta-Analysis (PRISMA) were applied [22]. The review protocol is available from the authors on request.

### Outcome definitions and recommended measurement methods

Definitions and criteria for ‘best practice’ screening methods for retinopathy, nephropathy and hypertension were sought. Detailed recommendations were available in American, Canadian and British guidelines [23–25]:

#### Diabetic retinopathy

The presence and characteristic evolution of typical retinal microvascular lesions in an individual with diabetes. Besides micro-aneurysms, blood vessel changes include intra-retinal haemorrhage, and vascular tortuosity and malformation (non-proliferative retinopathy) leading to abnormal vessel development (proliferative retinopathy). Seven-standard field stereoscopic-colour fundus photography with interpretation by a trained reader is the recommended standard screening for diabetic retinopathy, though direct ophthalmoscopy or indirect slit-lamp fundoscopy through dilated pupil or digital fundus photography may also be used. Treatment with laser photocoagulation surgery prevents vision loss [26–29]. The Canadian Diabetes Association Clinical Practice Guidelines Expert Committee [24] advocates that screening should be undertaken at least annually. However, the American Diabetes Association [23] advocates consideration of lesser frequency (every two - three years) following one or more normal eye examinations.

#### Nephropathy

A glomerular filtration rate (GFR) less than 60 mL/min present for three or more months, or any evidence of kidney damage for three or more months regardless of GFR [30]. In addition to any anatomical or pathological abnormalities or glomerular haematuria, it can be revealed by micro- or macroalbuminuria/proteinuria. Screening for nephropathy in adults with diabetes entails estimation of the level of kidney function and assessment of urinary albumin excretion. Significantly reduced kidney function is evidenced by an estimated GFR less than 60 mL/min; serum creatinine should be used to estimate GFR and stage the level of chronic kidney disease. Albuminuria should be determined through a timed/24-hour collection, or through a random spot test to determine albumin to creatinine ratio (ACR). The measurement of a spot urine for albumin, without simultaneously measuring urine creatinine, is susceptible to false negative/positive determinations.

Microalbuminuria was identified as urinary albumin excretion of either 30–299 or 300 mg/day in a 24-hour urine collection, with variations based on differing guidelines [23, 24], or an ACR of 2.0 - 20.0 mg/mmol. Macroalbuminuria (overt nephropathy) was identified as 300 mg/day or above if a 24-hour urine collection was performed, or an ACR of greater than 20.0 mg/mmol.

#### Blood pressure

Recommended targets are less than 130/80 mmHg for people with diabetes. Measurement of blood pressure should be undertaken by trained personnel, with participants in the seated position with feet on the floor and arm supported at heart level, after five minutes of rest. Cuff size must be appropriate for the arm circumference, with elevated values confirmed on a separate day. The American Diabetes Association [23] advocate that blood pressure should be measured at every routine visit.

### Literature search methods

MEDLINE (Ovid) and Scopus (which incorporates Embase journals), CINAHL, Science Direct (Elsevier), Google Scholar and Cochrane were searched by the first author to June 2014 to identify relevant articles. The MESH headings ‘Diabetes Mellitus, Type 1’; ‘Diabetic Retinopathy’; ‘Diabetic Nephropathies’; ‘Hypertension’; ‘Prevalence’; ‘Cross-sectional Studies’; and ‘Prospective Studies’, and keywords ‘Type 1 diabetes’; ‘Insulin Dependent Diabetes Mellitus’; ‘Juvenile Onset Diabetes Mellitus’; ‘Retinopathy’; ‘Eye Diseases’; ‘Nephropathy’; ‘Kidney Diseases’; ‘High Blood Pressure’; and Longitudinal Studies’ were used. The full search strategy can be viewed in Additional file 1. In addition, reference lists of all eligible studies were hand-searched.

Inclusion criteria were:

- Samples with type 1 diabetes;
- Mean age (plus 1SD) 18–30 years, or where the results for this age range were reported separately from other age groups; and
- English language studies only due to lack of resources for translation.

Exclusion criteria:

- Studies reporting data collected pre 1993 as from this date the definitive Diabetes Control and Complications Trial [9] established that the onset and progression of micro-vascular complications can be significantly reduced by HbA1c management. This changed diabetes management to make glycaemic control central, and hence management and complication rates may have changed.

### Search outcome

**Figure 1:**
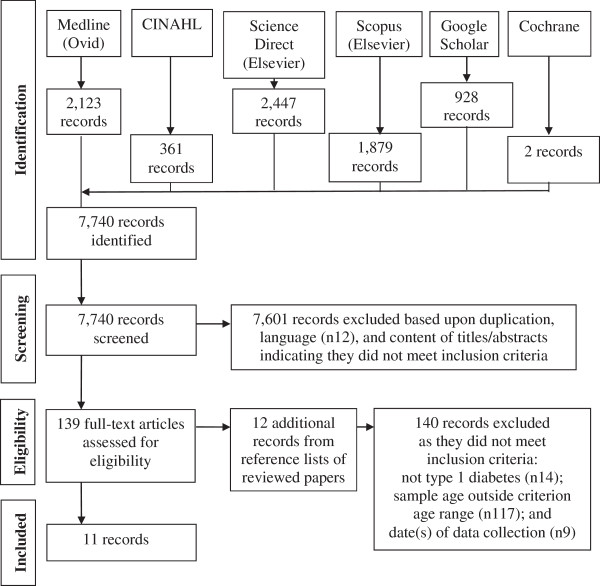
**Literature search and review flow chart.**

A total of 7,740 records were identified, downloaded to EndNote version X4 and screened by reading titles and abstracts. Of these, 7,601 records were excluded as duplicates or not meeting review inclusion criteria, including 12 non-English language papers. The remaining 139 full-text articles were assessed for eligibility; their reference lists were searched and an additional 12 papers identified. Of these 151 papers, 140 did not meet review inclusion criteria, leaving eleven relevant papers [31–41]. The search process and outcomes are summarised in Figure 1.

### Quality appraisal

With no universally accepted ‘gold standard’ method for evaluating and interpreting epidemiological study quality [42], to determine the strength of evidence quality appraisal was undertaken using the Strengthening the Reporting of Observational Studies in Epidemiology (STROBE) [43] checklist for cohort, case-control, and cross-sectional studies. Eligible papers were also evaluated for methods of assessment and measurement of retinopathy, nephropathy and hypertension in relation to current evidence-based guideline recommendations. This appraisal can be viewed in Additional file 2. To ensure reliability in data extraction and quality appraisal, a sample of papers included in the review were independently appraised and data extraction compared by the second and last authors (six papers each). Agreement was reached for all papers.

### Data extraction and synthesis

Data were extracted to a purpose-designed spread-sheet in Microsoft Office Excel based on relevant elements of the Consolidated Standards of Reporting Trials (CONSORT) checklist [44]. Extracted data can be viewed in Table 1 and Additional file 3. The number of diabetes centres involved in each study was noted to aid interpretation of transferability of findings.

**Table 1:** **Reported prevalence of diabetic retinopathy**

Author(s); country of origin	Number of centres	Age, mean (SD) yrs.; sample size	Any DR	Non-proliferative DR	Proliferative or treated DR	Predictors
**Arfken et al. 1998; U.S.A.** [31]	Not provided	19.0 (11.0) (White); n 215 (n 312 TS)			10.2%	Moderate/severe DR (proliferative DR)
						OR 12.40 (* 5.31 - 28.98)
						Moderate/severe DR in White participants (proliferative DR)
						OR 16.55 (* 5.43 - 50.45)
						HbA1c (2% change) (proliferative DR)
						OR 1.92 (* 1.36 - 2.70)
						HbA1c (2% change) in White participants (proliferative DR)
						OR 2.17 (* 1.34 - 3.50)
**Broe et al. 2014; Denmark** [32]	Not provided	21.0 (3.3) (In 1995 study), 20.2 (3.2) (Not in 1995 study); n 185 (In 1995 study), n 139 (Not in 1995 study) (n 324 TS)		In 1995 study: 61.2%	In 1995 study: 0.5%	
				Not in 1995 study: 51.8%	Not in 1995 study: 0.7%	
**James et al. 2014; Australia** [34]	Not provided	23.0 (3.7); n 95 (2010), n 85 (2011) (n 707 TS)	2010: 13.7% 2011: 9.4%			
**LeCaire et al. 2006; U.S.A.** [36]	Not provided	9 yrs.: 18.8 (7.2) (DR -), 21.1 (6.4) (DR +); n 290 (n 474 TS)	9 yrs.: 47%	9 yrs.: 33% (Min); 11% (M); 2% (Mod - Sev)	9 yrs.: 0.3%	Age at examination (per year) (DR)
						< 20 years Hazard ratio 1.2 (* 1.1 - 1.3)
						≥ 20 years Hazard ratio 1.0 (* 1.0 - 1.0)
						Diabetes duration at examination
						versus 4 years (DR)
						7 years Hazard ratio 1.6 (* 0.8 - 3.3)
						9 years Hazard ratio 4.1 (* 2.2 - 7.6)
						14 years Hazard ratio 7.9 (* 3.5 - 17.5)
						Non -White-race (versus White race)
						Hazard ratio 1.6 (* 0.8 - 3.0)
						HbA1c (per 1%) by diabetes duration (DR)
						4 years Hazard ratio 1.1 (* 1.0 - 1.3)
						7 years Hazard ratio 1.4 (* 1.3 - 1.6)
						9 years Hazard ratio 1.4 (* 1.2 - 1.6)
						14 years Hazard ratio 1.2 (* 1.0 - 1.6)
						Male sex (DR)
						Hazard ratio 1.3 (* 1.0 - 1.7)
**Olsen et al. 1999; Denmark** [37]	19 and five^	Median 21.1 (range 12.0 - 26.9); n 205 > 20 yrs. (n 339 TS)		48.9% (Min) 20% (Mod plus)		
**Olsen et al. 2004; Denmark** [38]	19 and six^	20.4 (3.2) (Prepubertal diabetes onset), 24.2 (1.3) (Pubertal/post-pubertal diabetes onset); n 304 (Prepubertal diabetes onset), n 49 (Pubertal/post-pubertal diabetes onset ) (n 353 TS)	57.6%			HbA1c (DR); p < 0.0001
						Diabetes duration
						before puberty (DR); p < 0.05
						after the onset of puberty (DR); p < 0.001
**Salardi et al. 2012; Italy** [40]	n 11	Very young pre-pubertal onset 22.0 (4.5); n 53 (n 105 TS)	40% Diabetes duration - < 20 yrs.: 27% > 20 yrs.: 88%	30% (M); 10% (Mod - Sev)		

(DR) Diabetic retinopathy (M) Mild (Min) Minimal (Mod) Moderate (n) Number (OR) Odds ratio (Sev) Severe (SD) Standard deviation.

(TS) Total sample (*) 95% Confidence interval (^) 19 paediatric departments and five/six departments of internal medicine.

## Results

The eleven papers derived from nine separate studies and mainly employed cross-sectional research designs; three papers that had provided data applicable to the target age group had involved a 1995 cohort of a Danish nationwide longitudinal study [32, 37, 38]. Data were collected via case note audit in three studies, and via documentation surveys in two further studies. Only three of the eleven studies solely provided data relating to the target population; all varied in their methodological quality and are summarised in Table 1 and Additional files 2 and 3. Ethnicity was reported in only three papers, and only one study focused on rural/non-urban populations or localities.

### Prevalence and prediction of retinopathy

The prevalence of diabetic retinopathy in the target population was reported in seven papers [31, 32, 34, 36–38, 40]. Prevalence data were reported for 215 applicable participants by Arfken et al. [31], for both 95 (2010) and 85 (2011) participants by James et al. [34], and 53 by Salardi et al. [40]. In longitudinal studies data were also reported on 324 applicable participants by Broe et al. [32], on 290 at nine years diabetes duration by LeCaire et al. [36], on 190 by Olsen et al. [37], and on 353 by Olsen et al. [38]. These two latter papers reported data from 19 paediatric departments (both) and five/six departments of internal medicine, whereas Salardi et al. [40] reported data from eleven centres; the other four papers did not provide detail.

Retinopathy was assessed and measured according to current best practice guideline recommendations in all papers. In these seven studies from four different countries with participants sampled by different methods, retinopathy prevalence varied somewhat (Table 1). Salardi et al. [40] reported an overall prevalence of 40%, with 27% and 88% at less than or greater than 20 years diabetes duration, respectively, whereas James et al. [34] reported a prevalence of 13.7% and 9.4%, and Olsen et al. [38] 57.6%; LeCaire et al. [36] reported 47% with retinopathy at nine years diabetes duration (6% - 73% with retinopathy at mean ages 19.5 - 24.8 years). At similar diabetes duration proliferative or treated diabetic retinopathy was reported in 10.2% of participants by Arfken et al. [31], but affected 0.3% of those at nine years diabetes duration by LeCaire et al. [36]; Broe et al. [32] reported a prevalence of proliferative retinopathy of 0.5% - 0.7%.

Data were provided relating to predictors of diabetic retinopathy in the target population in only two of these studies. Arfken et al. [31] reported that in White participants, a strong association was demonstrated between the development of proliferative retinopathy and existing moderate/severe diabetic retinopathy (Odds Ratio (OR) 16.55 (95% Confidence Interval (CI) 5.43 - 50.45)). Glycaemic control was also shown to be significant (2% change in HbA1c; OR 2.17 (95% CI 1.34 - 3.50)). This latter finding was consistent with Olsen et al. [38] who reported long-term glycaemic control (p < 0.0001) and diabetes duration before and after puberty onset (p < 0.05 and p < 0.001, respectively) as significantly associated with the development of retinopathy (Table 1). Other findings were generated from samples inclusive of but not specific to the target population.

### Prevalence and prediction of nephropathy

Prevalence of diabetic nephropathy was reported in eight papers [32–35, 37–40]. Raile et al. [39] reported data from 262 centres and, as previously reported, Salardi et al. [40] from eleven centres and both Olsen et al. [37] and Olsen et al. [38], from 19 paediatric departments (both) and five/six departments of internal medicine; the number of centres from which data were obtained was unclear in James et al. [34]. In all of these five papers renal function indices employed were not in accordance with current best-practice guideline recommendations. These were however utilised by Broe et al. [32] who reported 18 (10.5%) and 17 (14.8%) participants with albuminuria, and Garg et al. [33] who reported data from a single eye/kidney clinic. In a study involving 150 participants, 24 (16%) were reported with albumin excretion indicative of microalbuminuria and eleven (7.3%) with values indicative of macroalbuminuria. Prevalence data were also reported for 121 participants by Kullberg et al. [35]. For this study, neither number of study sites nor detail of study assessment methods for nephropathy were supplied. At recruitment for fundus photography sample ages ranged mean (SD) 12.4 (2.1) - 41.7 (2.4) years, with subgroups A3 aged 21.9 (2.2) years and A4 27.2 (2.3) years. In these subgroups 14% and 13% were reported with urinary albumin excretion greater than 20 mg/L. Factors predicting development of nephropathy were not reported by either Broe et al. [32] or Garg et al. [33].

### Prevalence and prediction of hypertension

The prevalence of hypertension was reported in five papers [33–35, 40, 41]. Schwab et al. [41] and Salardi et al. [40] reported data derived from 195 and eleven centres, respectively, but did not detail young adult cohort numbers. As previously noted, Kullberg et al. [35] reported prevalence data from 121 eligible participants but did not detail numbers of sites.

Criteria for hypertension in adults with diabetes were revised down to 130/80 mmHg earlier this century. All three papers reported the prevalence of hypertension either without stating diagnostic criteria or using what are now out-dated criteria (140/90 mmHg). Kullberg et al. [35] and Salardi et al. [40] reported hypertension by their definitions as occurring in 0% - 9% of participants. Schwab et al. [41] reported raised systolic and diastolic blood pressures in 11% and 2.6% of applicable participants, respectively, with 4.8% receiving pharmacotherapy. Out-dated criteria were also used by Garg et al. [33] who reported blood pressure values categorised by participants’ albumin excretion rate grouping. They reported 34% - 72.5% of systolic and 37.7% - 64.9% of diastolic ambulatory blood pressure measurements (mean of 24-hour collections) as above the 90% percentile of normal for age, gender and ethnic group. For participants with macroalbuminuria, over 60% of day and night-time systolic and diastolic measurements were above the 90th percentile of normal values.

Indices employed by James et al. [34] were in accordance with current best-practice guideline recommendations. Blood pressure measurements were documented in 313 and 306 of participants, with 33.9% and 30.7% having mean values within hypertensive ranges, respectively. With anti-hypertensive medication prescribed for 10.2% of participants a total of 201 (48.4%) were classified as hypertensive; at least one documented hypertensive measurement was reported in 35 (48.6%) cohort members prescribed anti-hypertensive medication, across the study period. Participants were more likely to have hypertension if they had no (rather than any) health service contact (OR 0.21, 95% CI 0.1 - 0.51, p = 0.001) or a longer diabetes duration (each year, OR 1.05, 95% CI 1.01 - 1.09, p = 0.006). This was in addition to use of continuous subcutaneous insulin infusion therapy (OR 1.8, 95% CI 1.2 - 2.7, p = 0.004) although this latter finding may have been affected by missing data.

## Discussion

This systematic literature review indicated that vascular complications are common amongst young adults with type 1 diabetes although the results reported varied somewhat. Some form of retinopathy occurred in up to almost half of participants; more severe forms affected up to one in ten. One in six was reported with microalbuminuria; one in 14 had macroalbuminuria. Hypertension occurred in almost one in two participants. In out-dated high thresholds this decreased to approximately one in ten participants. The frequency of these complications is concerning since they are largely preventable, are occurring alongside an increasing incidence of type 1 diabetes worldwide, and incur high costs in financial and health-related quality of life terms. Ng and Morlet [45] flagged the high prevalence and cost of diabetic retinopathy amongst Australians, but failed to differentiate the particular problems of younger onset and hence greater lifetime burden for those with type 1 diabetes. The DiabCo$t Australia study [46] estimated the minimum annual cost of type 1 diabetes in Australia at between $430 to $570 million in 2008, with expenditure increasing with the presence of complications. Real costs were acknowledged as higher, as costs associated with disability and premature mortality were not considered.

Identified prevalence rates of retinopathy in this young adult population were elevated compared to recent data for adolescents with type 1 diabetes. Downie et al. [47] reported a prevalence of 12% between 2005–2009, compared to up to 40% and 57.6% in the literature reviewed here [38, 40]. The review rate was not dissimilar to rates provided for older cohorts of people with type 1 diabetes (within a decade outside the review age criteria). Karadeniz and Yilmaz [48], Esteves et al. [49] and Roy [50] reported retinopathy prevalence of 33.2%, 44.4% and 63.9%, respectively; discrepancies perhaps reflected the trend of increasing prevalence of complications with increasing diabetes duration and age.

In studies where data were obtained using current best practice recommendations, prediction of development of nephropathy was not reported for the young adult age group. Studies of older cohorts of people with type 1 diabetes found diabetic nephropathy associated with indices of diabetes duration and control (increasing HbA1c), and with prevalence and severity of other forms of vascular disease and population-wide markers of vascular risk such as triglyceride levels and weight [51–55].

The identified prevalence rates of hypertension in this young adult population were also elevated compared to a study involving a slightly older cohort (mean (SD) age 33.8 (11.8) years at baseline), which reported an increase in elevated systolic and diastolic blood pressures over time. In 2003–2004, 17.9% and 6%, respectively, were affected, whereas by 2006–2007 this had increased to 28.8% and 8.2%. The proportion of participants prescribed anti-hypertensive medication also increased significantly during this period, from 20.7% to 34.2% [56]. However, in another similarly older cohort (mean (SD) age 37 (9) years) only 48% of those diagnosed and treated for hypertension achieved target values [57], indicating little room for complacency. This is consistent with review findings.

The paucity of blood pressure data for young adults and the indication of poor achievement of treatment goals are particular concerns. Hypertension predisposes to stroke, myocardial infarction, cardiac failure and limb amputation as well as other vascular disease manifestations such as retinopathy and nephropathy. A trend seen in slightly older young adults with type 1 diabetes was of any one end-organ manifestation of vascular disease indicating an increased likelihood of concurrent vascular disease in other areas. For example, in cohorts with mild/severe renal failure, 71.4% and 83.3%, respectively, also had hypertension [58]. Early detection and prompt treatment are therefore essential, with general population studies clearly demonstrating early diagnosis and adherence to treatment prevents or delays development and progression of end-organ damage [59].

Adherence to sometimes complex, always life-long medication schedules is challenging. ‘Typical’ versus ‘ideal’ medication adherence in patients with hypertension has demonstrated nearly double the relative risk of myocardial infarction, angina and stroke [60]. However, Hill et al. [61] cited achievement of up to 80% adherence rates in routine care and this is especially important for this patient group as cardiovascular disease occurs more than ten times more frequently in those with type 1 diabetes than in age-matched non-diabetes populations [62]. Lack of data on the prevalence of hypertension may hamper prioritisation and appropriate targeting of therapy; important opportunities for treatment may be missed.

Effective prevention interventions rely on identifying modifiable predictors of vascular complications. Data relating specifically to the target population were scarce and this quantitative epidemiological systematic review found glycaemic control as predictive of vascular disease in young adults with type 1 diabetes. Diabetes duration was also flagged, of concern because it is not modifiable and almost half of those who develop the disease do so before age 15 years, many in infancy and childhood [63]. After only nine years with type 1 diabetes almost half of young adults had retinal damage [36] - and probably other vascular disease as well.

On the other hand glycaemic control is modifiable and influential. The deterioration that accrues with disease duration may be ameliorated by better glycaemic control [9, 64], with better control being achieved by those who maintain contact and relationships with their diabetes healthcare teams [65]. This flags the crucial importance of ensuring that services are able to support young adults with type 1 diabetes, particularly during the vulnerable period when they leave the paediatric services that supported them as children, establish relationships with adult services and independent self-management practices. It reinforces the importance of regular screening using best practice methods as this offers the best chance for early detection and initiation of appropriate treatment, and consequently to minimise visual loss and blindness, renal failure and dialysis, heart failure and strokes occurring in young adults.

Good quality data are required from adequately powered studies to inform service development, to help nurses and other healthcare professionals risk-stratify and provide appropriate support to young adults with type 1 diabetes, to minimise and defer onset of vascular complications. In most developed countries the data required for high-powered studies are collected routinely by diabetes services. That these data have not been accessed and used to develop algorithms to stratify risk for these young adults is indicative of the lack of priority accorded this problem.

Some limitations apply to the current review. A search for grey literature such as conference abstracts was not undertaken; neither were experts in this field contacted for unpublished data, nor authors for data from age-specific subsets where these data did not appear in publications. Identification of a specific age range to designate ‘young adults’ was challenging; we opted to focus on those who would have transitioned out of paediatric into adult care, but use of wider age ranges may have yielded additional data.

Caution also needs to be exercised when considering how review findings can be generalised to the target population of young adults with type 1 diabetes as few studies focused solely on representative samples of this specific age group or involved rural populations. Other omissions were the paucity of studies undertaken in developing countries, and limited data indicating participants’ ethnicity. Finally, although studies reporting data collected pre 1993 were excluded in light of the definitive Diabetes Control and Complications Trial [9], it may have taken a number of years for these research findings to change practice such that glycaemic control became central in every-day management. Earlier literature reviewed may therefore be poorly representative of current practice and not reflect prevalence of vascular complications in today’s young adults. New primary research is required.

## Conclusion

This is the first systematic review of the prevalence and predictors of retinopathy, nephropathy and hypertension in young adults with type 1 diabetes. While data were limited, underlying vascular disease manifesting as retinopathy and hypertension was common amongst this group, with development predicted by glycaemic control - and probably diabetes duration. With only one of these two factors amenable to clinical management, findings have implications for clinicians, policy-makers, patients and families: to raise the priority of improving glycaemic control as a means to defer and avoid development of complications which otherwise appear near-inevitable.

Clinical messages of this review are the importance of prevention of loss to follow up and provision of appropriate support, particularly around the vulnerable transition period from paediatric to adult-based care. This would ensure support for optimal glycaemic control and enable regular complication screening to be implemented - essential for early detection and treatment in this age group. Quality data are required to be available to clinicians and patients to stratify risk and guide treatment planning, and to inform service development. The message for policy-makers is that the prevalence rates identified make good preventive care essential. The challenge is to make this a realistic option and available to all young adults with type 1 diabetes.

## Author information

SJ - RN, CDE, *Doctor of Philosophy (Nursing) student, Faculty of Health, University of Technology, Sydney.* RG - PhD, MN, RN, *Professor of Nursing, Charles Perkins Centre/Sydney Nursing School, University of Sydney.* JD - PhD, BAg, *Research Assistant, University of Newcastle.* LP - PhD, MSc, RN, *Professor of Nursing Research, and Practice Development, Faculty of Health, University of Technology, Sydney and Prince of Wales Hospital/Sydney and Sydney Eye Hospitals.*

## Electronic supplementary material

Additional file 1: **Search strategy.** (PDF 18 KB)

Additional file 2: **Strengthening the reporting of observational studies in epidemiology (STROBE) checklist.** (PDF 31 KB)

Additional file 3: **Summary of extracted information from included literature.** (PDF 70 KB)
