# Supplementary material for: Prevalence of vascular complications and factors predictive of their development in young adults with type 1 diabetes: systematic literature review
Source: BMC Res Notes. 2014 Sep 2;7:593. doi: 10.1186/1756-0500-7-593 (PMC4167503; doi:10.1186/1756-0500-7-593)
Supplement: Supplementary file 1 — Additional file 1: Search strategy. (PDF 18 KB) [file 13104_2014_3143_MOESM1_ESM.pdf]

Additional file 1: Search strategy.

Type 1 diabetes

1. ‘Diabetes Mellitus, Type 1’; or ‘Type 1 diabetes’; or ‘Insulin Dependent Diabetes Mellitus’; or ‘Juvenile Onset Diabetes Mellitus’

Vascular complications

2. ‘Diabetic Retinopathy’; or ‘Retinopathy’; or ‘Eye Diseases’
3. ‘Diabetic Nephropathies’; or ‘Nephropathy’; or ‘Kidney Diseases’
4. ‘Hypertension’; or ‘High Blood Pressure’
5. 2 OR 3 OR 4

Prevalence

6. ‘Prevalence’; or ‘Cross-sectional Studies’; or ‘Prospective Studies’; or ‘Longitudinal Studies’

Summary

7. 1 AND 5 AND 6
