# Supplementary material for: Prevalence of vascular complications and factors predictive of their development in young adults with type 1 diabetes: systematic literature review
Source: BMC Res Notes. 2014 Sep 2;7:593. doi: 10.1186/1756-0500-7-593 (PMC4167503; doi:10.1186/1756-0500-7-593)
Supplement: Supplementary file 2 — Additional file 2: Strengthening the reporting of observational studies in epidemiology (STROBE) checklist. (PDF 31 KB) [file 13104_2014_3143_MOESM2_ESM.pdf]

Additional file 2: Strengthening the reporting of observational studies in epidemiology (STROBE) checklist

	Arfken et al. 1998	Broe et al. 2014	Garg et al. 1997	James et al. 2014	Kullberg et al. 2002	LeCaire et al. 2006	Olsen et al. 1999	Olsen et al. 2004	Raile et al. 2007	Salardi et al. 2012	Schwab et al. 2006
**Title and abstract**	√	√	√	√	√	√	√	√	√	√	√
Introduction											
**Background/ rationale**	√	√	√	√	√	√	√	√	√	√	√
**Objectives**	√	√	√	√	√	√	√	√	√	√	√
Methods											
**Study design**	√		√	√	√	√	√	√	√	√	√
**Setting**	√	√	√	√	√	√	√	√	√	√	√
**Participants**	√	√	√	√	√	√	√	√	√	√	√
**Variables**	√	√	√	√	√	√	√	√	√	√	√
**Data sources/ measurement**	√	√	√	√	√	√	√	√	√	√	√
**Bias**	√	X	√	X	X	√	X	X	X	√	X
**Study size**	√	√	X	√	√	√	√	√	√	X	√
**Quantitative variables**	√	√	√	√	√	√	√	√	√	√	√

**Statistical methods**	√	√	√	√	√	√	√	√	√	√	√
Results											
**Participants**	N/A	√	√	√	N/A	√	X	√	N/A	√	N/A
**Descriptive data**	√	√	√	√	√	√	√	√	√	√	√
**Outcome data**	√	√	√	√	√	√	√	√	√	√	√
**Main results**	√	√	√	√	√	√	√	√	√	√	√
**Other analysis**	√	√	√	√	√	√	√	√	√	√	X
Discussion											
**Key results**	√	√	√	√	√	√	√	√	√	√	√
**Limitations**	√	√	X	√	X	√	X	X	X	√	X
**Interpretation**	√	√	√	√	√	√	√	√	√	√	√
**Generalisability**	√	√	X	√	√	√	√	√	X	X	√
Other information											
**Funding**	√	√	√	√	√	√	√	X	√	X	√
Assessment and measurement according to best practice guidelines											
**Diabetic retinopathy**	√	√	N/A	√	N/A	√	√	√	N/A	√	N/A
**Nephropathy**	N/A	√	√	X	X	N/A	X	X	X	X	N/A
**Hypertension**	N/A	N/A	X	√	X	N/A	N/A	N/A	N/A	X	X

(N/A) Not applicable
