# Supplementary material for: Prevalence of vascular complications and factors predictive of their development in young adults with type 1 diabetes: systematic literature review
Source: BMC Res Notes. 2014 Sep 2;7:593. doi: 10.1186/1756-0500-7-593 (PMC4167503; doi:10.1186/1756-0500-7-593)
Supplement: Supplementary file 3 — Additional file 3: Summary of extracted information from included literature. (PDF 70 KB) [file 13104_2014_3143_MOESM3_ESM.pdf]

Additional file 3: Summary of extracted information from included literature.

**Aims/purpose/research question; study design; setting; data collection dates; sampling strategy; sample size and characteristics**	**Method(s) of complication assessment**	**Relevant findings (excluding data on predictors)**
**Arfken et al. 1998**		
To compare the risk of developing proliferative DR in African-American and White participants with type 1 diabetes<br><br>Cross-sectional design; case note audit<br><br>U.S.A.; ‘model demonstration units’, number unclear<br><br>Data collection period unclear<br><br>Sampling strategy unclear; inclusion criteria: subjects with type 1 diabetes (age of onset of ≤ 40 years, continuous insulin usage); African-American or White; at least 2 visits with gradable eye photographs; if > 2 visits, visits chosen to maximise follow-up duration<br><br>n 312 (n 97 (African-American participants); n 215 (White participants))<br><br>*Age: 27.0 (15.0) years (African-American participants); 19.0 (11.0) years (White participants); $p = 0.0001$<br>Male: 32% (African-American participants); 45% (White participants); $p < 0.03$<br>*Diabetes duration: 9.2 (7.0) years (African-American participants); 8.0 (6.4) years (White participants); $p < 0.15$	**DR**<br>Photography	**DR**<br>Proliferative: 17.5% (African-American); 10.2% (White participants)

**Broe et al. 2014**		
To investigate the long-term incidence of proliferative DR, and progression and regression of DR and associated risk factors in young Danish patients with type 1 diabetes<br><br>Longitudinal cohort study<br><br>Denmark; number of centres unclear<br><br>Selection of participants from an earlier study. 16-year follow-up examination data in 2011 compared with participants' baseline 1995 data (the latter shown here). Mean age not reported for 2011 follow-up<br><br>n 324 (n 185 (participants from baseline 1995 study); n 139 (non-participants from baseline 1995 study))<br><br>Baseline (1995) characteristics:<br>*Age: 21.0 (3.3) years (participants from baseline 1995 study); 20.2 (3.2) years (non-participants from baseline 1995 study); $p < 0.03$<br><br>*Diabetes duration: 13.5 (3.3) years (participants from baseline 1995 study); 13.0 (2.9) years (non-participants from baseline 1995 study); $p = 0.22$	**DR**<br>Photography<br><br>**Nephropathy**<br>Two consecutive overnight timed urine samples<br><br>Microalbuminuria:<br>AER 20 - 200 µg/min<br>Macroalbuminuria:<br>AER >200 µg/min	**DR**<br>*Participants from 1995 study -*<br>Non-proliferative: 61.2% (n 114)<br>Proliferative: 0.5% (n 1)<br><br>*Non-participants from 1995 study -*<br>Non-proliferative: 51.8% (n 72)<br>Proliferative: 0.7% (n 1)<br><br>**Nephropathy**<br>*Participants from 1995 study -*<br>Albuminuria (mean AER > 20 µg/min): 10.5% (n 18)<br><br>*Non-participants from 1995 study -*<br>Albuminuria (mean AER > 20 µg/min): 14.8% (n 17)<br><br>**HT**<br>*Participants from 1995 study -*<br>Antihypertensive treatment: 5.8% (n 10)<br><br>*Non-participants from 1995 study -*<br>Antihypertensive treatment: 6.9% (n 9)

**Garg et al. 1997**		
To determine the relationship between 24-hour ambulatory BP measurements and early renal disease<br><br>Cross-sectional design<br><br>U.S.A.; 1 eye/kidney clinic<br><br>Data collection period unclear<br><br>Consecutive sampling; inclusion criteria: subjects with type 1 diabetes who had completed the 24-hour ambulatory BP measurements, brought in two timed overnight urine specimens and who attended the clinic were included; exclusion criteria: subjects with a body-mass index greater than 120% of normal for their age and gender<br><br>n 150 (n 86 (normal AER); n 29 (borderline AER elevation); n 24 (microalbuminuria); n 11 (macroalbuminuria))<br><br>*Age: 22.6 (3.3) years (normal AER 22.7 (0.5) years; borderline AER elevation 21.3 (0.6) years; microalbuminuria 23.0 (0.6) years; macroalbuminuria 24.3 (1.0) years)<br>Male: 51.3% (normal AER 52.3%; borderline AER elevation 51.7%; microalbuminuria 45.8%; macroalbuminuria 54.6%)<br>*Diabetes duration: 12.8 (5.0) years (range, 3.5 - 25.8) (normal AER 12.8 (0.6) years; borderline AER elevation 11.1 (0.8) years; microalbuminuria 13.7 (1.0) years; macroalbuminuria 15.0 (1.8) years)	**HT**<br>Ambulatory BP measurements were taken by an oscillometric portable automatic monitor every 30 minutes from 6 a.m. to 10 p.m. and every hour from 10 p.m. to 6 a.m.; readings were downloaded.<br>Office BPs were measured using the appropriate sized cuff and a sphygmomanometer after resting in sitting position for 5 minutes<br><br>HT > 140/90 mmHg<br><br>**Nephropathy**<br>Overnight urine collections taken on nights with no evening exercise, alcohol or caffeine intake and when menses, pregnancy, or urinary tract infections were absent<br><br>Borderline elevation:<br>AER 7.6 - 20 µg/min<br>Microalbuminuria:<br>AER 20.1 - 200 µg/min<br>Macroalbuminuria:	**HT**<br>% 24-hour ambulatory BP measurements indicating -<br>Systolic HT: borderline AER elevation 12.3% (2.8); microalbuminuria 6% (1.8); macroalbuminuia 40.2% (7.6); $p < 0.0001$<br>Diastolic HT: borderline AER elevation 11.1% (2.6); microalbuminuria 7.8% (1.5); macroalbuminuria 39.3% (8.8); $p < 0.0001$<br><br>% of ambulatory BP measurements > 90% percentile (mean of 24-hours) -<br>Systolic: borderline AER elevation 48.3% (5.1); microalbuminuria 37.8% (4.9); macroalbuminuria 72.5% (8.4); $p < 0.0002$<br>Diastolic: borderline AER elevation 47.3% (4.3); microalbuminuria 40.5% (4.1); macroalbuminuria 64.9% (10.5), $p < 0.002$

	AER > 200 µg/min	
**James et al. 2014**		
To identify the prevalence and factors predictive of development of vascular complications in a cohort of young adults with type 1 diabetes<br><br>Cross-sectional design; case note audit<br><br>Australia; number unclear<br><br>Data collected 2010 - 2011<br><br>Participants accessing Hunter New England Local Health District public health services, identified through clinic records, hospital attendances and other clinical records<br><br>n 707 (n 682 (2010); n 707 (2011))<br>Ophthalmic examinations documented: n 95 (2010); n 85 (2011)<br>ACR measurements documented: n 222 (2010); n 218 (2011)<br>BP measurements documented: n 313 (2010); n 306 (2011)<br><br>*Age: 23.0 (3.7) years<br>Male: 54.3%<br>*Diabetes duration: 10.2 (5.8) (range 0.2 - 28.3) years<br>Aboriginal and/or Torres Strait Islander 5.6%; Rural participants 42.4%	**DR**<br>Documented<br><br>**Nephropathy**<br>≥ one reported ACR measurement above laboratory threshold normal value<br><br>**HT**<br>≥ 130/80 mmHg per annum, and/or prescription of anti-hypertensive medication	**DR**<br>*2010* -<br>Any: 13.7%<br><br>*2011* -<br>Any: 9.4%<br><br>**Nephropathy**<br>*2010* -<br>≥ one ACR measurement above laboratory threshold value: 15.1%<br><br>*2011* -<br>≥ one ACR measurement above laboratory threshold value: 16.1%<br><br>≥ two above threshold value: 12.4%<br><br>**HT**<br>*2010:*<br>≥ 130/80 mmHg: 33.9%<br><br>*2011:*<br>≥ 130/80 mmHg: 30.7%

		Anti-hypertensive medication: 10.2%<br><br>Any HT: 48.4%
**Kullberg et al. 2002**		
To investigate the prevalence and incidence of vascular complications in a population with type 1 diabetes from a well-defined geographical area<br><br>Cross-sectional design; case note audit<br><br>Sweden; number of centres unclear<br><br>1994 - 1995<br><br>Total n 390 (n 258 (age at diabetes onset 0 - 19 years); n 132 (age at diabetes onset 20 - 35 years))<br><br>Consecutive sampling from registers from local diabetes centres; inclusion criteria: type 1 diabetes; diagnosed < 36 years of age, during 1983 - 1987, and at the time of onset living within a defined geographical area - consecutive cases<br><br>Grouped by age at diagnosis: A3 (10 - 14 years) n 75; A4 (15 - 19 years) n 46.<br><br>*Age at recruitment/fundus photo:<br>A3 21.9 (2.2) years; A4 27.2 (2.3) years<br>Male: A3 56%; A4 61%	**DR**<br>Photography<br><br>**Nephropathy**<br>Measured with the standard (unstated) method at each clinic<br><br>**HT**<br>> 140/90 mmHg or on anti-hypertensive medication(s)	**DR**<br>Data not provided specific to target age group<br><br>*Age at diabetes onset 0 - 19 years -*<br>Microaneurysms: 23.3%<br>> than microaneurysms: 3.9%<br><br>*Age at diabetes onset 20 - 35 years -*<br>Microaneurysms: 21.2%<br>> than microaneurysms: 11.4%<br><br>**Nephropathy**<br>UAE > 20 mg/L: A3 14%; A4 13%<br><br>**HT**<br>Any HT: A3 9%; A4 9%

*Diabetes duration at fundus photo: A3 9.4 (1.8) years; A4 9.8 (1.6) years		
**LeCaire et al. 2006**		
To examine development of DR in a population-based cohort of persons with incident type 1 diabetes, to investigate the possibility of lowered DR prevalence and severity compared with previous U.S. studies<br><br>Longitudinal cohort study<br><br>U.S.A.; number of centres unclear<br><br>Voluntary recruitment to cohort with inclusion criteria: type 1 diabetes diagnosed from May 1987 - April 1992; ≤ 30 years of age; living within defined area in Southern and Central Wisconsin<br><br>n 474 (n 420 (4 years diabetes duration (T1)); n 275 (7 years diabetes duration (T2)); n 290 (9 years diabetes duration (T3)); n 68 (14 years diabetes duration (T4)))<br><br>*Age: T1 14.1 (6.2) years (DR -); 19.5 (7.0) years (DR +) ($P \leq 0.0001$). T2 16.1 (6.6) years (DR -); 19.5 (6.4) years (DR +) ($P \leq 0.01$). T3 18.8 (7.2) years (DR -); 21.1 (6.4) years (DR +) ($P \leq 0.01$). T4 22.2 (8.2) years (DR -); 24.8 (6.3) years (DR +)<br>Male: T1 51% (DR -); 52% (DR +). T2 49% (DR -); 48% (DR +). T3 49% (DR -); 57% (DR +). T4 39% (DR -); 46% (DR +)<br>Ethnicity (White) T1 96% (DR -); 96% (DR +). T2 96% (DR -); 90% (DR +). T3 99% (DR -); 95% (DR +). T4 100% (DR -); 98% (DR +)	**DR**<br>Photography	**DR**<br>Any: T1 6%; T2 23%; T3 47%; T4 73%<br>Minimal non-proliferative: T1 5%; T2 18%; T3 33%; T4 44%<br>Mild non-proliferative: T1 1%; T2 4%; T3 11%; T4 19%<br>Moderate - severe non-proliferative: T1 0.2%; T2 0.4%; T3 2%; T4 10%<br>Proliferative or treated: T1 0%; T2 0.4%; T3 0.3%; T4 0%

**Olsen et al. 1999**		
To estimate the prevalence of present glycaemic control and the prevalence of microvascular complications in a cohort of children and adolescents who had participated in 2 previous studies<br><br>Longitudinal cohort study<br><br>Denmark; 19 paediatric departments and five departments of internal medicine<br><br>Selection of participants from two previous studies (1987 and 1989)<br><br>Study n 339 (n 205 > 20 years of age of which n 190 assessed for DR, and n 192 assessed for nephropathy); Median age 21.1 years (range 12.0 - 26.9), male 53.1% and duration 13.2 years (8.9 - 24.5). n and characteristics of sample > 20 years not reported	**DR**<br>Photography<br><br>**Nephropathy**<br>Two consecutive overnight timed urine samples. If AER was > 20 µg/min in one of the two samples a third sample was collected. The mean of 2 consistent AER samples was used in the analaysis<br><br>Microalbuminuria:<br>AER of 20 - 150 µg/min<br>Macroalbuminuria:<br>AER >150 µg/min	*Age > 20 years:*<br><br>**DR**<br>Minimal non-proliferative: 48.9%<br>Moderate non-proliferative plus: 20%<br><br>**Nephropathy**<br><br>Microalbuminuria: 9.4%<br>Macroalbuminuria: 4.7%
**Olsen et al. 2004**		
To determine the effect of the pre-pubertal duration of diabetes on early DR and elevated AER<br><br>Longitudinal cohort study	**DR**<br>Photography	**DR**<br>Any: 57.6%

Denmark; 19 paediatric departments and six departments of internal medicine<br><br>Selection of participants from an earlier study. Eight year follow-up data (1995 - 1996)<br><br>n 353 (n 304 (Onset of diabetes < 12 years (pre-pubertal); n 49 (Onset of diabetes ≥ 12 years (pubertal/postpubertal)))); n 339 had urine samples taken<br><br>*Age: 20.4 (3.2) years (Onset of diabetes < 12 (pre-pubertal)); 24.2 (1.3) years (Onset of diabetes ≥ 12 years (pubertal/post-pubertal)); p < 0.0001<br>Male: 51.3% (Onset of diabetes < 12 years (pre-pubertal)); 65.3% (Onset of diabetes ≥ 12 years (pubertal/post-pubertal))<br>*Duration: 13.8 (3.2) years (Onset of diabetes <12 years (pre-pubertal)); 10.7 (1.3) years (Onset of diabetes ≥ 12 years (pubertal/post-pubertal)); p < 0.0001)	**Nephropathy**<br>Two out of three consecutive overnight timed urine samples<br><br>Microalbuminuria:<br>AER 20 - 150 µg/min<br>Macroalbuminuria:<br>AER >150 µg/min	**Nephropathy**<br>AER > 20 µg/min: 12.7%
**Raile et al. 2007**		
To analyse the prevalence of nephropathy in a nationwide prospective survey<br><br>Prospective cross-sectional design, documentation survey<br><br>Germany and Austria; 262 centres<br><br>Data collection period unclear but ceased February 2007	**Nephropathy**<br>Measurement of ACR in a random spot collection, 24-hour collection with creatinine, or timed (e.g. overnight) collection.<br><br>Microalbuminuria or macroalbuminuria was defined as at least two increased urine albumin	**Nephropathy**<br>Microalbuminuria: 3.3%<br>Macroalbuminuria: 0.2%<br>End stage renal disease: 0.8%

Sample from German Diabetes Documentation System with inclusion criteria of at least 2 documented urine analyses; strategy unclear<br><br>n 27,805 (n 26,644 (normal). n 919 (microalbuminuria); n 52/229 (macroalbuminuria/end stage renal disease)<br><br>*Age at last visit: 21.1 (0.1) years (normal); 28.7 (0.6) years (microalbuminuria); 37.2 (1.2) years (macroalbuminuria/ end stage renal disease); $p < 0.0001$<br>Male: 52.6% (normal); 52.1% (microalbuminuria); 58% (macroalbuminuria/end stage renal disease)<br>*Diabetes duration: 8.3 (0.05) years (normal); 12.6 (0.4) years (microalbuminuria); 20.1 (0.9) years (macroalbuminuria/ end stage renal disease); $p < 0.0001$	tests during the follow-up<br><br>Microalbuminuria: AER 20 - 199 µg/min or an urinary albumin creatinine ≥ 2.5 mg/mmol<br>Macroalbuminuria: AER ≥ 200 µg/min or an urinary albumin creatinine ≥ 35 mg/mmol	
**Salardi et al. 2012**		
To compare the effects of the pre-pubertal duration of diabetes on the occurrence of complications in two groups of patients after the same number with years of the disease<br><br>Cross-sectional design<br><br>Italy; 11 centres<br><br>2007 - 2009<br><br>Patients initially diagnosed and treated between 1981 - 1992, those who were aged 0 - 3 years and those who were in puberty or post-pubertal at the onset of type 1 diabetes; obtained from individual centres but	**DR**<br>Photography	**DR**<br>*Entire cohort -*<br>Any after 20 years diabetes duration: 55%<br>Mild after 20 years diabetes duration: 40%<br>Moderate non-proliferative after 20 years diabetes duration: 9%<br>Severe non-proliferative after 20 years diabetes duration: 4%<br>Proliferative after 20 years diabetes duration: 2%

sampling strategy unclear<br><br>n 105 (n 53 (very young pre-pubertal onset); n 52 (pubertal onset)); n 86 assessed for UAE; n 89 assessed for HT<br><br>*Age: 22.0 (4.5) years (very young pre-pubertal onset); 31.6 (4.1) years (pubertal onset)<br>Male: 43% (41.5% (very young pre-pubertal onset); 44.2% (pubertal onset))<br>*Diabetes duration: 19.7 (4.0) (range 15 - 28.5) years; n 69 (< 20 years); n 36 (≥ 20 years)		*Very young pre-pubertal-onset group -*<br>Any: 40%<br>Mild: 30%<br>Moderate to severe: 10%<br>Any < 20 years diabetes duration: 27%<br>Any > 20 years diabetes duration: 88%<br><br>*Pubertal onset group -*<br>Any: 71%<br>Mild: 52%<br>Moderate to severe: 20%<br>Any < 20 years diabetes duration: 63%<br>Any > 20 years diabetes duration: 63%
	**Nephropathy**<br>UAE or AER -<br>Microalbuminuria: UAE 30 - 300 mg/day or AER ≥ 20 µg/min<br>Macroalbuminuria: UAE > 300 mg day or AER > 150 µg/min	**Nephropathy**<br>*Entire cohort -*<br>Abnormal UAE: 7%<br><br>*Very young pre-pubertal-onset group -*<br>Abnormal UAE: 4%<br><br>*Pubertal onset group -*<br>Abnormal UAE: 9%
	**HT**<br>BP was measured using a standard sphygmanometer with patients seated, and calculated as the mean of two measurements	**HT**<br>*Entire cohort -*<br>Any: 3%<br><br>*Very young pre-pubertal-onset group -*<br>Any: 0%

	HT: > 140/90 mmHg	*Pubertal onset group -*<br>Any: 7%
**Schwab et al. 2006**		
To ascertain the type and prevalence rate, age and sex distribution of cardiovascular risk factors in type 1 diabetic patients up to 26 years of age<br><br>Cross-sectional design, documentation survey<br><br>Germany and Austria; 195 centres<br><br>2003 - 2004<br><br>Sampled consecutive cases from a joint-national register; inclusion criteria: type 1 diabetes.<br><br>n 27,358 (n 25,184 assessed for raised systolic BP; n 25,178 assessed for raised diastolic BP; n 27,358 assessed for HT treatment)<br>Cohort divided into pre-pubertal (0.25 - 11 years), pubertal (12 - 16 years) and young adulthood (17 - 26 years) based upon developmental stage<br>Size of each cohort unclear<br><br>*Age: 7.5 (2.5) years (pre-pubertal); 13.7 (1.4) years (pubertal); 18.5 (2.3) years (young adulthood) (P < 0.0001)<br>Male: 51.7% (pre-pubertal); 51.7% (pubertal); 52.5% (young adulthood) (P value NS)	**HT**<br>Use of a sphygmanometer. Median value calculated from at least three measurements<br><br>HT: Average systolic or diastolic BP ≥ to the 95th percentile for age and sex. Values not provided for adults	**HT**<br>Systolic: 8.1%<br>Diastolic: 2.5%<br>Raised systolic BP: 5.8% (pre-pubertal); 7.4% (pubertal); 11% (young adulthood); p < 0.0001<br>Raised diastolic BP: 3.9% (pre-pubertal); 3.2% (pubertal); 2.6% (young adulthood); p < 0.0001<br>Receiving anti-hypertensive medication: 2.1% (0.2% (pre-pubertal); 1.4% (pubertal); 4.8% (young adulthood)); p < 0.0001

*Diabetes duration: 2.5 (2.3) years (pre-pubertal); 4.9 (3.6) years (pubertal); 8.2 (4.8) years (young adulthood) (P < 0.0001)		

(ACR) Albumin-creatinine ratio (AER) Albumin excretion rate (BP) Blood pressure (DR) Diabetic retinopathy
(HT) Hypertension (n) Number (OR) Odds ratio (P) Probability (UAE) Urinary albumin excretion *Mean (SD)
